# Brain Functional Connectivity Significantly Improves After Surgical Eradication of Porto-Systemic Shunting in Pediatric Patients

**DOI:** 10.3390/life15020290

**Published:** 2025-02-13

**Authors:** Gianvincenzo Sparacia, Giuseppe Parla, Roberto Miraglia, Jean de Ville de Goyet

**Affiliations:** 1 Radiology Service, BiND, University of Palermo, 90128 Palermo, Italy; 2 Radiology Service, IRCCS-ISMETT, 90127 Palermo, Italyrmiraglia@ismett.edu (R.M.); 3 Department for the Treatment and Study of Pediatric Abdominal Diseases and Abdominal Transplantation, IRCCS-ISMETT, 90127 Palermo, Italy; jdeville@ismett.edu

**Keywords:** minimal hepatic encephalopathy, non-cirrhotic portal hypertension, Abernethy malformation, Meso-Rex bypass, magnetic resonance imaging, resting-state functional magnetic resonance imaging, connectomics

## Abstract

Purpose: Porto-systemic shunting (PSS) in patients with Abernethy malformation (AM) or obstruction of the portal vein (OVP) is often associated with normal liver parenchyma and hepatic function. This association provides an interesting natural model for studying the brain functional connectivity changes secondary to PSS but independently from hepatic (dys)function. Because PSS can be eliminated with appropriate interventions, these particular conditions offer a unique physio-pathological model where the same patient can be studied in both “active PSS” and “absent PSS” conditions (pre- and post-cure analyses). Methods: Four children (<18 years) who were evaluated for Abernethy malformation (n = 2) or portal cavernoma (n = 2) and underwent corrective surgery (living-donor liver transplantation for AM, or Meso-Rex bypass for OPV, respectively) were included in the study. Brain magnetic resonance imaging and resting-state functional magnetic resonance imaging (rest-fMRI) were acquired in all patients before and after the corrective surgery. A functional connectome analysis was performed before (“active PSS” condition) and after (“absent PSS”—physiological condition) the cure of PSS. Results: As a result of the cancelation of PSS, rest-fMRI connectomics revealed a statistically significant (*p* < 0.05 family-wise error) improvement in global brain functional connectivity in both groups following each surgical procedure. Conclusions: In this clinical model of isolated PSS (with absence of hepatic dysfunction), brain functional connectivity was altered even in young patients and in the absence of hyperammonemia; moreover, specific interventions to cancel out PSS consequently significantly improved brain functional connectivity.

## 1. Background

In the vast majority of patients presenting with porto-systemic shunting (PSS), the primary cause is liver disease with cirrhosis and secondary portal hypertension: they thus present with a combination of hepatic dysfunction (HD) and PSS. Both HD and PSS alter the brain metabolism and activity—a potent synergistic association resulting in a spectrum of neurological dysfunctions that is complex to study, such as of mixt etiology. A typical neuro-clinical presentation is “hepatic encephalopathy” (HE)—a condition that co-exists in a variable manner (quantitatively and qualitatively) with impairment of cognitive functions, attention disorders, gross disorientation, motor system abnormalities, and possible neuropsychiatric disturbances [1,2,3]. The presence and the magnitude of PSS is a key factor for the development of HE. It is a common clinical observation and also suggested by (1) the impairment of brain function after the medical creation of PSS [4,5,6], and (2) the rapid improvement of brain activity and of cognitive functions after closure of PSS by liver transplantation, for example [7,8]. Although recent findings support the role of inflammation (systemic or cerebral) as a complementary or synergistic processus [9,10], hyperammonemia has been pointed out for decades as a key factor for the development of HE, and still remains the first metabolic element in the chain of pathological events [11,12,13]. Ammonia easily crosses the blood–brain barrier, passively or via transporters, and is preferentially captured by astrocytes; though astrocytes are equipped precisely for ammonia detoxification, excess of ammonia leads rapidly to oxidative stress, mitochondrial fragmentation, cell dysfunction, and neurotransmitter imbalance. These alterations in turn lead to astrocyte swelling and senescence, microglial cell activation, neuronal damage and death, and osmotic gliopathy [13,14,15,16].

**Figure 1:**
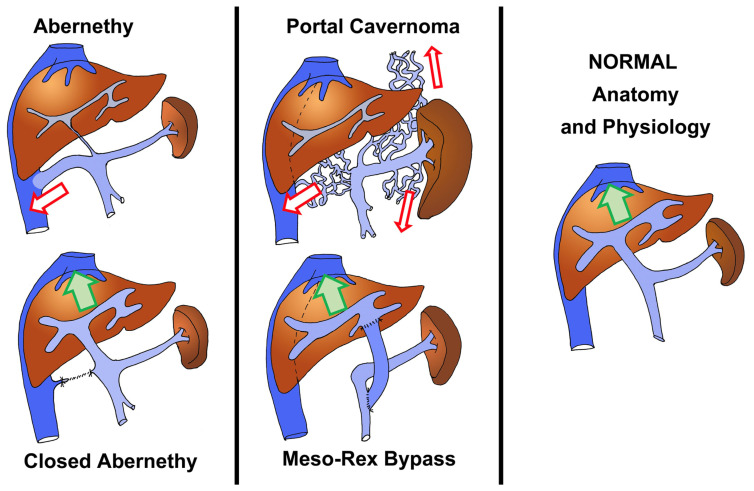
Graphical representations of Abernethy malformation (left column) and portal cavernoma (middle column): the lower figures show the condition after a surgical cure (liver replacement in Abernethy patients, and Meso-Rex bypass in cases with portal cavernoma). The normal physiologic condition is represented in the right column. The arrows indicate the direction of main portal flow (normal flow: green arrows; porto-systemic shunting: red arrows).

In the majority of cases, PSS is secondary to portal hypertension due to liver disease, but there are a few specific conditions combining PSS and normal liver parenchyma/hepatic function. This is found in most patients with (1) a congenital malformation known as Abernethy malformation (AM) (the latter consists of a large communication between the portal system and the systemic venous circulation), and (2) extrahepatic portal hypertension secondary to the obstruction of the portal vein (OPV) (Figure 1) [17,18,19,20].

OPV [17,18,19,20] is a prevalent cause of chronic portal hypertension in infants. Thrombosis of the portal vein results in a cavernous transformation of the hepatic hilum, also known as portal cavernoma [17] (Figure 1). In the absence of hepatic dysfunction (the liver parenchyma is usually normal), the natural progression of the disease is characterized by repeated hemorrhage in childhood and the development of splenomegaly and hypersplenism [1]. As portal hypertension progresses, there is increasing porto-systemic shunting (PSS) due to the development of retro-peritoneal, parietal, and para-esophageal venous neo-collaterals. AM is a serious condition characterized by a congenital PSS and its collateral effects [21,22] (Figure 1). The shunts can be located within or outside the liver and can reroute either part or the whole splanchnic blood flow into the systemic circulation; though the liver can be altered histologically with time, patients typically do not experience liver dysfunction in the pediatric age range [21,22].

The presence of a PSS in patients with normal hepatic function brings an interesting natural model to study the collateral effects of PSS on the functioning of the brain, in the absence of hepatic dysfunction. More interesting is that in these conditions (AVM and OPV), the PSS can be closed by precise interventions—which eventually restores a normal physiology. This is a unique opportunity for studying the physio-pathology of PSS in a natural human model, comparing the condition both before and after the cure, in the same patient. Because of the absence of associated liver dysfunction in both AM and OVP, ammonia blood levels are minorly or mildly elevated [3,6,10,11,12]. Although overt HE is rare in infants of these groups, they may present a less aggressive form of HE, called “covert” or “minimal hepatic encephalopathy” (MHE) [22,23,24].

While HE has been well studied in cirrhotic patients for decades, MHE in OPV is just starting to attract interest for clinical research, with studies confirming that MHE is a clinical reality that may affect overall quality of life and even patients’ ability to perform daily tasks [25,26,27]. Although MHE is usually clinically subtle (neurological examination may be normal), it may be characterized by a range of quantifiable cognitive impairments, psychomotor sluggishness, attention deficits, and reduced fine motor skills [17,18,24]. The condition impacts often on quality of life, and may alter to various degrees the neurocognitive development, scholar and academic performance, and even the carrying out of long-term problems of socio-professional integration and psychiatric condition. Psychometric and electrophysiological evaluations are often unable to diagnose MHE in otherwise asymptomatic patients. Incidence is high, with MHE conditions affecting 30 to 50% of OVP and AM cases [23,24,25,26].

The standard brain imaging may be normal or showing only slight abnormalities such as low-grade edema, or a T1-weighted-hyperintense globus pallidus [27]. The precise physio-pathology of T1-weighted hyperintensity is not clear: though the deposition of manganese, lipids, or other compounds that possess paramagnetic properties has been evoked [28], hyperintensity may be secondary to a toxic-ischemic insult or metabolic disorder and reflect only local edema or inflammation—both processes being considered key elements of the HE physio-pathological mechanism. In that, T1-weighted hyperintensity of the globus pallidus may be easy imaging suggesting some brain alteration relating to PSS. Interestingly, it has been clearly shown that this anomaly is reversible after a PSS cure, by liver transplantation, for example [7,8]. Although neurological symptoms may be minimal or absent, microstructural brain abnormalities and low-grade edema have been evidenced by standard MR in patients with MHE, but these findings are far too little for modern research on HE/MHE [29,30].

Advanced MRI techniques have been used to capture more minor changes or study the metabolism (MR spectroscopy, metabolite study, diffusion tensor imaging, 3D-T1, …), but the research in that matter has remained limited with little new relevant conclusions [4,15,31,32,33]. Most of the latter studies differ from our research in that most studied (1) the progression of HE/MHE before and after PSS creation, and not the contrary; (2) cirrhotic patients with severe hepatic dysfunction, not MHE in patients with normal hepatic function; and (3) using MR spectroscopy and metabolite changes rather than functional connectivity. In a recent study, Yadav et al. studied both cirrhotic and OPV patients (including MR diffusion tensor imaging and spectroscopy, but no MR connectivity study), and confirmed that OPV patients have less severe MHE compared to cirrhotic patients—a predictable conclusion [32].

Rest-fMRI has been used extensively in the last decade to study brain functionality, showing that it can identify MHE/HE, and possibly detect anomalies even before the onset of true MHE [4,34,35]. However, Wang et al. confirmed the limit of these rest-fMRI studies [31], as their conclusions are based on the assumption that functional connectivity is stable—when the current opinion is, on the contrary, that (1) it is highly dynamic and (2) MHE features are precisely alterations of the dynamics. Wang et al. concluded that further research should be towards dynamic functional connectivity [31].

At a similar but higher level, the “Human connectome project” was launched in 2010 and, aiming at studying brain connectivity from the nematode to the human, has been challenged by the vastness of the field and the magnitude of the task [36]. Even the simple rest-fMRI studies have been limited in number and application. Assessment of brain connectivity by rest-fMRI has not until now, as far as we know, been used to study children with PSS.

Interestingly, for both AM and OPV, there is a possible cure by rerouting the splanchnic venous blood towards the liver and closing the PSS: this allows the restoration of the hepatic clearance of ammonia and reinstates a normal hepatic and systemic physiology. Restoring the portal blood flow to the liver and eliminating the PSS has been shown by Chiu et al. and Mack et al., being able to reverse hyperammonemia and HE and improving neurocognitive impairment in patients with OPV; similarly, Franchi-Abella et al. mention that HE secondary to AM is cured by closure of PSS [37,38].

For OVP children, the Meso-Rex bypass (MRB) procedure [19,20] (Figure 1) restores the portal flow towards and through the liver by interposing a vascular conduit between the superior mesenteric vein and the recessus of Rex (intrahepatic left portal vein). It is a physiological cure of OPV, restoring normal portal pressure and normal splanchnic venous physiology, with the disappearance of esophageal varices and PSS; it has thus become the preferred method of treatment for OPV when feasible. For AM children, the preferred approach consists of closing the PSS early in life (Figure 1) in order to prevent the systemic effects of PSS on the developing brain of the infant (alteration of neurocognitive development, minimal encephalopathy). For those cases that cannot benefit from closure, liver transplantation with closure of the PSS is indicated when they develop complications [22].

The objective of this research was thus to investigate the feasibility of using rest-fMRI for assessment of the brain connectivity dysfunction secondary to PSS. This has never been tried in similar clinical settings, as far as we know. The hypothesis was that this tool would be more sensitive, and specific, compared to other, currently available, assessment tools. Moreover, because the patients were exposed to closure of PSS, a unique opportunity was given to study the brain functioning in a longitudinal manner (same patients)—thereby possibly confirming the reversibility of initial functional alterations.

## 2. Material and Methods

### 2.1. Patient Population and Selection Criteria

All children (<18 years) who were assessed for OPV or AM in the past 5 years at our center were considered for the study. Of these children, only those who had undergone a brain rest-fMRI before and after a corrective procedure were then selected for the study.

### 2.2. Study

The study was conducted retrospectively and written informed consent was obtained from the parents or legal guardians of all selected children, for the retrospective use of clinical and radiological data. All clinical, laboratory, and radiological data and procedures, including MRI imaging, were acquired for clinical purposes and as part of the usual assessment or follow-up.

Rest-fMRI was performed only in patients who had an independent clinical indication for standard MRI before the intervention (i.e., studying the venous drainage of the brain before the resection of one internal jugular vein in OVP patients, or assessing signs of HE in neurologically pauci-symptomatic AM cases). Functional MRI was performed as a complementary study during MRI. In the latter patients, a brain rest-fMRI was repeated after the operation: consent was obtained, based on the indication of re-assessing the brain function after the preoperative fMRI showed lower-than-expected activity.

Based on their diagnosis and the surgical procedure used to correct the condition, patients were divided into two groups: group 1 consisted of AM children undergoing living-donor liver transplantation (LDLT), and group 2 consisted of children with OPV undergoing Meso-Rex bypass correction.

The primary endpoint was to determine if there was a statistically significant difference in functional connectivity between baseline and post-treatment assessments in each of the two groups within one year after surgery.

### 2.3. Statistical Analysis

Statistical analyses were carried out using the CONN toolbox’s built-in statistical functions [21].

### 2.4. Imaging Analysis

MRI examinations were performed on a 3T MRI scanner (Discovery 750w, General Electric Medical System, Milwaukee, WI, USA) utilizing a 32-channel head coil. Participants were positioned in the scanner with their heads comfortably restrained by foam padding to reduce head movement. Earplugs were used to reduce the noise of the scanner. During the resting-state scans, participants were instructed to keep their eyes closed and remain as motionless as possible and were asked not to think about any specific thoughts or perform any specific, rhythmic mental activity.

Standard multi-parametric MR imaging protocol:

MR imaging was performed with fast spin echo (FSE) T1-weighted and T2-weighted MR images, fluid-attenuated inversion recovery (FLAIR), T2*-weighted gradient-recalled echo (GRE), susceptibility weighted imaging (SWI), and standard 3-direction diffusion weighted imaging (b = 0, b = 1000). Additionally, isotropic T1-weighted volumetric imaging (3D-SPGR) was used to assess cortical gray matter volume changes. To assess cerebral network connectivity (functional MRI), rest-fMRI with the blood oxygenation level-dependent (BOLD) echo-planar imaging (EPI) technique was performed to assess spontaneous neuronal activity within the resting-state networks (RSNs), resulting in a total of 210 volumes.

### 2.5. Data and Functionnal Image Pre-Processing

Data pre-processing was conducted using a dedicated Linux-based workstation [https://ubuntu.com/ accessed on 12 February 2022] and using various software tools for connectomics computation, including Freesurfer [http://surfer.nmr.mgh.harvard.edu/ accessed on 12 February 2022], fMRIPrep [https://fmriprep.org/en/0.6.6/index.html accessed on 12 February 2022], Statistical Parametric Mapping (SPM 12) [https://www.fil.ion.ucl.ac.uk/spm/ accessed on 12 February 2022], and CONN toolbox [https://web.conn-toolbox.org/ accessed on 12 February 2022] [39] (running under MatLab (MATLAB version: 9.13.0 (R2022b), MathWorks Inc, Natick, MA, USA)).

For signal stabilization, the initial 10 volumes of the functional time series were discarded, with the first volume recorded to account for head movement. All participants’ rotations and movements were within 2 mm in the x, y, and z planes. Volumes were normalized to the standard EPI template in Montreal Neurological Institute (MNI) space [https://brainmap.org/training/BrettTransform.html accessed on 12 February 2022] and restored to 3 × 3 × 3 mm^3^. Spatial smoothing was obtained using a Gaussian kernel with a full width at half maximum of 8 mm. Subsequently, the CompCor function [40] was utilized for spatial and temporal pre-processing to reduce the influence of motion and physiological noise factors, as well as to identify and eliminate confounds in the BOLD signal. Additionally, first-order derivative terms for the whole brain, ventricular, and white matter signals were regressed as part of the correlation pre-processing to reduce the impact of spurious variance on neuronal activity. The rest-fMRI post-processing and analysis pipeline utilized was previously described [41].

The analysis of graph theory was used to create a mathematical model of the brain’s complex network functions. RSNs are represented by groups of nodes connected by edges [42]. As a result, the graph theory analysis illustrates the relationship between nodes and edges and depicts these interactions using various graph parameters (such as the clustering coefficient). Various RSNs in the brain, including the salience network, auditory network, basal ganglia network, higher visual network, visuospatial network, default mode network, language network, executive network, pre-cuneus network, primary visual network, and sensory-motor network, can be identified using these functional connectivity analysis techniques [42].

### 2.6. Seed Selection and Functional Connectivity Analysis

Assessment of the functional connectivity matrix in selected seed areas in RSNs was carried out by a region of interest (ROI) to ROI connectivity matrix comparison and by testing hypotheses [39]. Data visualization was obtained using the CONN toolbox. Functional connectivity measures were computed between seed areas for ROI-to-ROI analysis to identify patterns of ROI-to-ROI connectivity. Specifically, threshold-free cluster enhancement (TFCE) analyses [42,43] were used to make inferences at the cluster level. This was carried out within the framework of ROI-to-ROI connection matrices. TFCE analyses employ an automated hierarchical clustering method that utilizes optimal leaf ordering for hierarchical clustering [43,44]. This method is based on anatomical proximity or functional similarity metrics between regions of interest (ROIs). The analysis involves calculating a TFCE score for each T or F statistic associated with all voxels within the ROIs.

The TFCE score combines the strength of the statistical effect for each connection with the extent of all clusters or groups of neighboring connections that exhibit areas of functional activity. The objective of this method is to improve signal regions that indicate spatial continuity by eliminating the need for an arbitrary threshold to define the cluster.

Due to the extremely small number of patients and observations in this study, the distribution functions of the population from which the sample is drawn were unknown. To address this, a permutation method predicated on the assumption of interchangeability was used [42,43,44]. This method involves computing a test statistic by subjecting all subjects to multiple random permutations. Statistical inference is then conducted on the spatial maps associated with the various subjects using a non-parametric permutation statistical test [42,43,44] as previously described in a precedent study based on a single-subject analysis [45]. This test integrates the threshold-free cluster enhancement (TFCE) algorithm.

Functional connectivity between the two groups at baseline and follow-up after LDLT or Meso-Rex surgery was assessed using two-tailed paired *t*-tests. The resulting statistical maps were set with *p* < 0.05 at the cluster level, family-wise error (p-FWE) [46] being corrected. For maps that did not meet this criterion, a *p* < 0.001 threshold level was used.

### 2.7. Behavioral Correlations

To investigate changes in functional connectivity in the two groups before and after treatment (LDLT or Meso-Rex bypass), we conducted a correlation analysis using graph theory-based methodologies. Data were considered statistically significant if p-FWE corrected < 0.05. The data are presented as mean differences in effect size for RSN connectivity before and after each treatment for both groups.

The correlation coefficients, specifically the correlation matrix [39], were used to examine the increase in functional connectivity among the two groups of patients. The Conn toolbox utilizes Fisher transformed correlation coefficients, also known as Fisher transformed z values. The values provided are the inverse hyperbolic tangent values corresponding to the correlation coefficient. As a result, it is expected that the transformation will improve the normality of the data, thereby increasing the robustness of subsequent statistical analyses.

## 3. Results

Out of a cohort of around 70 children who were followed for portal hypertension and AM in the last decade in our unit, 4 patients complied with the selection criteria and were included in this analysis. In all four patients, surgery was indicated on the basis of conventional strategies: in the first group, AM was complicated by HE in one child and by an unresectable malignant tumor (hepatoblastoma) in the other case—and in the second group, both patients had portal hypertension and a favorable anatomy for a Meso-Rex bypass.

Two children (7 and 8 years of age, 20 and 24 Kg of weight) had AM, while two other children (7 and 9 years of age, 20 and 33 Kg of weight) had OPV. The preoperative ammonium blood level was normal in three cases, and was moderately increased in a single case—being 58 mic.mole/L (normal: 11–32 micr.mole/L). Only one child (AM) had a history of HE before the intervention; although the neurological examination was normal, he was referred after he had episodes of clinical encephalopathy in the past months, in the context of hyperammonemia.

**Figure 2:**
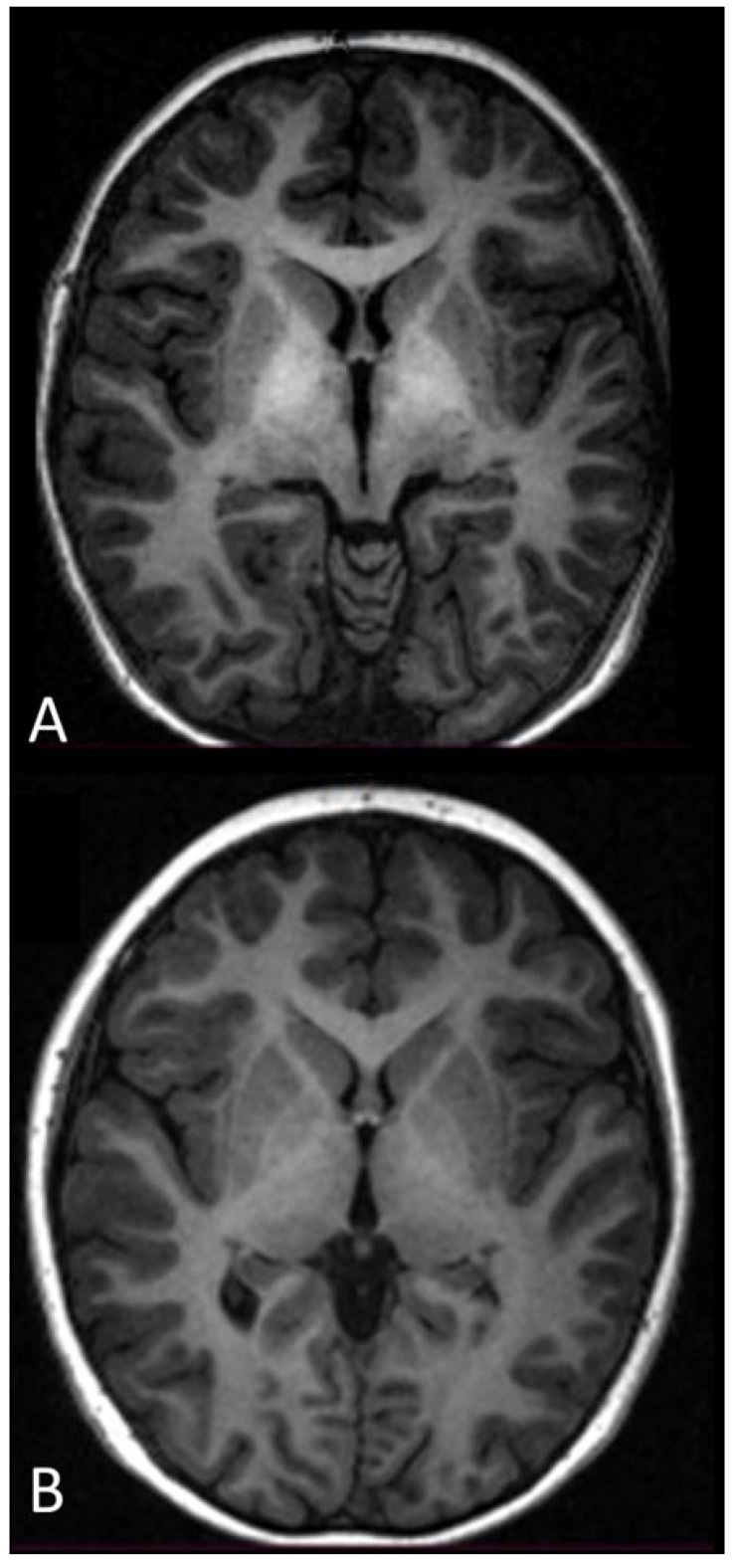
Axial T1-weighted MR image at the level of the basal ganglia of the brain (**A**) before surgery and (**B**) after surgery. (**A**) Bilateral symmetric hyperintensity in the globus pallidus on the T1-weighted MR image was observed in all cases in this series. (**B**) Bilateral symmetric hyperintensity in the globus pallidus on the T1-weighted MR image disappeared in the two patients who had follow-up >12 months.

Brain rest-fMRI was fully acquired in all four subjects within 3 months before their operation; the postoperative MRI was performed at a mean follow-up of 12.5 months after the procedure (interval: 5 to 22 months). Before the operation, hyperintensity of the globi pallidi was observed in the T1-weighted sequence in all four cases; this hyperintensity was still observable in two patients (one in each group) who had the postoperative control MRI within the first year, but was not seen any more in the two other patients who repeated the MRI within the second year (Figure 2).

**Figure 3:**
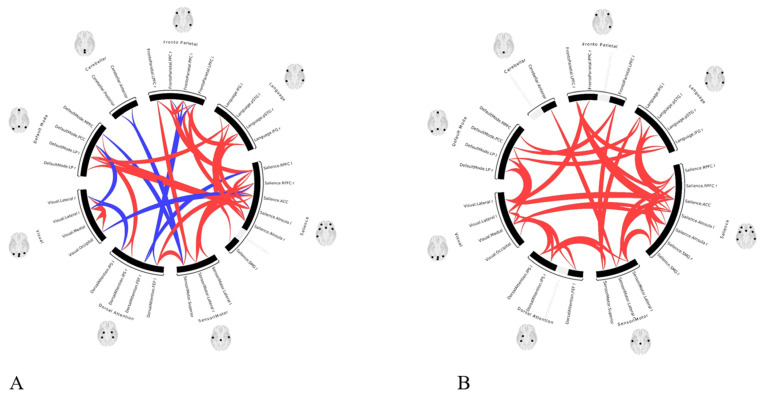
ROI-to-ROI color coded circular graphs show one-sample T-test statistics of the brain connectivity in group 1 patients (**A**) before LDLT and (**B**) after LDLT. There was a statistically significant (*p* < 0.05 FWE) increase in functional connectivity in the default mode, frontoparietal, salience, and sensorimotor networks in group 1 following LDLT. (ROI = Region of interest, LDLT = Living-donor liver transplantation, FEW = Family-wise error, In the color coded circular graph, the edges represent connectivity changes between regions; red connections mean positive T-values indicating increase in functional connectivity; blue color connections mean negative T-values indicating decrease in functional connectivity.

**Figure 4:**
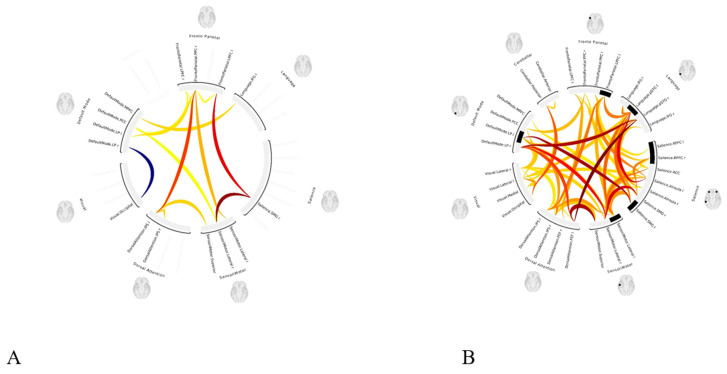
ROI-to-ROI color coded circular graphs show one-sample T-test statistics of the brain connectivity in group 2 patients (**A**) before Meso-Rex bypass and (**B**) after the procedure. Following Meso-Rex bypass, there was statistically significant increase in functional connectivity (*p* < 0.05 FWE) in default mode, frontoparietal, salience, and sensorimotor networks in group 2. (ROI = Region of interest, FEW = family-wise error, In the circular graph, the edges represent connectivity changes between regions, the colors correspond to network affiliation).

**Figure 5:**
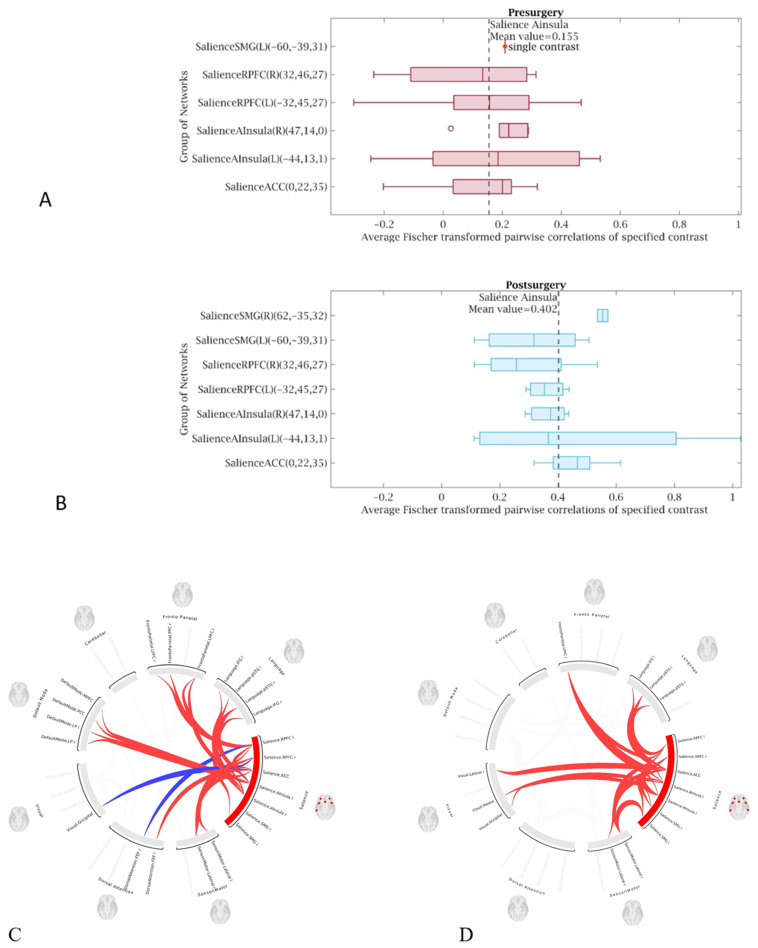
Visual representation of the increased functional connectivity throughout the salience network in patient’s group 1 after LDLT compared to the presurgical condition. The graphs showincreased mean value correlation coefficients (z values transformed using Fisher) in patient (**B**) after LDLT procedure (mean value 0.40) compared to (**A**) presurgical condition (mean value 0.15). (**C**,**D**) ROI-to-ROI color coded circular connectomes show increased functional connectivity throughout the salience network for patients in group 1 after LDLT (**D**) compared to the presurgical condition (**C**). (ROI = Region of interest, LDLT = living-donor liver transplantation, In the color coded circular graph, the edges represent connectivity changes between regions; red connections mean positive T-values indicating increase in functional connectivity; blue color connections mean negative T-values indicating decrease in functional connectivity.

**Figure 6:**
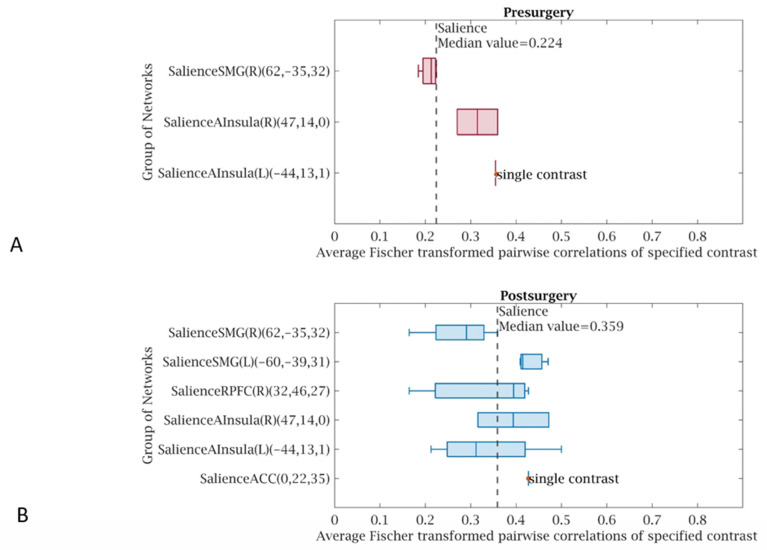

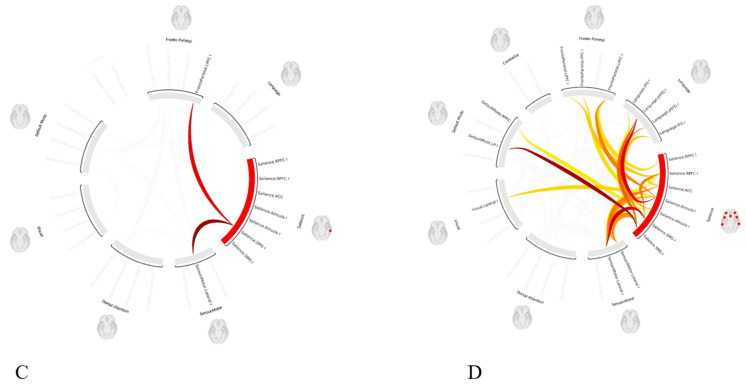
Visual representations of increased functional connectivity throughout the salience network for patients in group 2 after the Meso-Rex bypass compared to the presurgical condition. The graphs show increased median value correlation coefficients (z values transformed using Fisher) throughout the salience network (**B**) after the Meso-Rex bypass (median value 0.35) compared to the (**A**) presurgical condition (median value 0.22). (**C**,**D**) Color coded circular connectomes show increased functional connectivity throughout the salience network after the Meso-Rex bypass (**D**) compared to the presurgical condition (**C**). (In the circular graph, the edges represent connectivity changes between regions, the colors correspond to the network affiliation).

A statistically significant (*p* < 0.05 FWE) increase in functional connectivity was observed in the default mode, frontoparietal, salience, and sensorimotor networks as compared to the pre-treatment functional connectivity in the same networks, in group 1 following LDLT (Figure 3), and in group 2 after the Meso-Rex bypass (Figure 4). All children showed a highly significant improvement in the salience network after operation. Figure 5 and Figure 6 show comprehensive visual representations of the observed increase in correlation coefficients (z values transformed using Fisher) and functional connectivity across the salience network for both groups following each surgical treatment.

## 4. Discussion

The observations in this short series are original as no previous study in the literature has described similar findings. It is, as far as we know, the first research using rest-fMRI for studying brain connectivity in children with PSS.

The key findings were

1. Pre-intervention rest-fMRI showed low functional connectivity in all patients, though three of four were asymptomatic and were as young as 7 to 9 years old. It suggests that the brain functional alteration starts very early in the story of PSS or portal hypertension, much earlier than it has ever been thought.
2. Pre-intervention low functional connectivity was evidenced even in children with a normal ammonia blood level and normal liver function: this suggests that other factors or metabolites—not identified clearly until now—may play a role in the physio-pathogenesis of HE.
3. The functional connectivity did improve significantly after the correction of PSS, in all four patients—each case showing improvement compared to their own preoperative examination. This may impose revisiting our management strategies and opting for early cures rather than palliative or conservative management.
4. Though the phenomenon was slower, MRI evidenced the disappearance of the hyperintensity of the globus pallidus on T1-weighted sequences (in two cases that had follow-up >1 year). This needs further studying.

Some of these observations may have profound implications, for example, the observation that brain connectivity was significantly altered in patients with a normal ammonia blood level. It suggests that ammonia may not be the key element, but a second event: its potent toxicity against astrocytes and neuronal networks may be amplified by the fact that it acts as a second cellular hit, possibly synergistically. One candidate for the role of a “first hit” when hyperammonemia is absent could be—as evoked frequently in the literature recently—a neuroinflammatory process induced by peripheral inflammation [9,47]. One of the current hypotheses for the peripheral inflammatory signaling is a gut microbiome alteration secondary to portal hypertension, and induction of a pathological gut–brain axis signaling [48,49]. The observation by Zhand et al., and by Gitto et al., that an altered microbiome did improve after decreasing portal pressure in patients with severe portal hypertension is interesting but might be a new egg–chicken challenge [50,51].

There are several limitations to our study. First is the small number of pediatric cases in this series, which is a result of the rarity of these diseases. Although our unit is part of a tertiary hospital dedicated to complex surgery and transplantation, and although the unit follows a relatively large cohort of children with either AM or OVP, only four cases could be selected as the criteria imposed not only having a cure of the PSS, but also having a rest-fMRI before the procedure—which is not part of our standard assessment. Secondly, the results of rest-fMRI in this series could not be compared to that of healthy subjects, as this information is currently not available in the literature, or in our center. The latter limitation was, however, overcome, as all four patients were studied before and after the correction of PSS and each one was used as his own control. The statistical analysis method was based on mutual information and surrogate analysis, as described in a previous study [27], with an additional permutation method based on the assumption of interchangeability to compensate for the small number of pediatric cases and the lack of controls [24,25,26]. As a result, each patient serves as a self-reference for longitudinal connectomics analysis as a member of the group. A third limitation is that fMRI was performed with no other advanced study methodology such as metabolic MRI, MRI spectroscopy, or ultra-high-field MRI: this was not intended at all, as using fMRI in this study was allowed since it is a rapid and non-invasive technology that was added to an MRI examination for other purposes. The study was explorative in its concept, but it now opens the way to other research.

The rapid development of neuroscience in the last decades led to an increasing understanding of how the brain develop in infants and children, consisting not only of a major growth within the first 18–24 months of life, but also and more importantly of a slower maturation of the functional brain connectome from birth to adolescence, in relation to the neurocognitive development [52,53]. Moreover, there are specific times in a child’s development when certain functions are active but later disappear, with no possibility of reacquiring them later. Overall, this emphasizes how crucial it is to reconsider some of the medical strategies, with the priority of protecting at best the brain modeling and neurocognitive acquisitions during the first 10 years of life. In line with this objective, it is critical to provide early intervention for curing any condition that may affect the brain development in children [29]. In the context of natural or congenital PSS, this means prioritizing early intervention to cure the condition whenever possible in infancy. In the same line of thinking, these observations should stimulate rethinking the role of elective PSS creation (i.e., in the context of the management of chronic portal hypertension in children), in order to minimize the risk of favoring MHE. This would mean postponing indications to older age [54], and/or considering earlier pre-emptive liver replacement instead of opting for a palliative PSS.

## 5. Conclusions

As the primary objective of this research, the study confirmed the feasibility of using RS-fMRI for evidencing alterations of functional connectivity relating to HE or MHE. It also shows that it can be used in relatively young children, aged less than 10 years old. These two observations, per se, open a vast space for research in the future, and considering rs-fMRI as a new tool for early diagnoses, enable fine assessment of neurological collateral effects and monitoring the effects of the clinical management in children with portal hypertension and/or PSS.

Further studies are needed to confirm these observations. While they are preliminary observations, they cannot be ignored and could stimulate revisiting current strategies and approaches for managing OBV and AM patients, possibly also for dealing with a larger spectrum of PSS in the context of liver diseases causing chronic portal hypertension.

## Data Availability

Upon motivated request and within limitations imposed by privacy, access to data may be considered.

## Abbreviations

AM	Abernethy malformation
AUROC	Area under the receiver operating characteristic curve
fMRI	Functional magnetic resonance imaging
FSE	Fast spin echo
FOV	Field of view
FEW	Family-wise error
HE	Hepatic encephalopathy
LDLT	Living-donor liver transplantation
MELD	Model for end-stage liver disease
MHE	Minimal hepatic encephalopathy
MR	Magnetic resonance
MRB	Meso-Rex bypass
MRI	Magnetic resonance imaging
OVP	Obstruction of the portal vein
PSS	Porto-systemic shunting
rest-fMRI	Resting-state functional magnetic resonance imaging
ROI	Region of interest
ROC	Receiver operating characteristic
RSN	Resting-state network
SD	Standard deviation
TFCE	Threshold-free cluster enhancement

## References

[1] Häussinger D., Dhiman R.K., Felipo V., Görg B., Jalan R., Kircheis G., Merli M., Montagnese S., Romero-Gomez M., Schnitzler A. (2022). Hepatic encephalopathy. Nat. Rev. Dis. Primers.

[2] Lu K. (2023). Cellular Pathogenesis of Hepatic Encephalopathy: An Update. Biomolecules.

[3] Ridola L., Faccioli J., Nardelli S., Gioia S., Riggio O. (2020). Hepatic Encephalopathy: Diagnosis and Management. J. Transl. Int. Med..

[4] Qi R., Zhang L.J., Zhong J., Wu S., Zhang Z., Zhong Y., Ni L., Zheng G., Jiao Q., Wu X. (2012). Dynamic changes of intrinsic brain activity in cirrhotic patients after transjugular intrahepatic portosystemic shunt: A resting-state FMRI study. PLoS ONE.

[5] Häussinger D., Butz M., Schnitzler A., Görg B. (2021). Pathomechanisms in hepatic encephalopathy. Biol. Chem..

[6] López-Cervantes M., Quintanar-Stephano A., Alcauter-Solórzano S., Hernández-Pando R., Aguilar-Roblero R., Gasca-Martínez D., Ortíz J.J., Vázquez-Martínez O., Ximénez-Camilli C., Díaz-Muñoz M. (2021). Cerebellar spongiform degeneration is accompanied by metabolic, cellular, and motor disruption in male rats with portacaval anastomosis. J. Neurosci. Res..

[7] Cheng Y. (2017). Longitudinal Intrinsic Brain Activity Changes in Cirrhotic Patients before and One Month after Liver Transplantation. Korean J. Radiol..

[8] Hopp A.E., Dirks M., Petrusch C., Goldbecker A., Tryc A.B., Barg-Hock H., Strassburg C., Klempnauer J., Weissenborn K., Pflugrad H. (2019). Hepatic Encephalopathy Is Reversible in the Long Term After Liver Transplantation. Liver Transpl..

[9] Cabrera-Pastor A., Llansola M., Montoliu C., Malaguarnera M., Balzano T., Taoro-Gonzalez L., García-García R., Mangas-Losada A., Izquierdo-Altarejos P., Arenas Y.M. (2019). Peripheral inflammation induces neuroinflammation that alters neurotransmission and cognitive and motor function in hepatic encephalopathy: Underlying mechanisms and therapeutic implications. Acta Physiol..

[10] Aldridge D.R., Tranah E.J., Shawcross D.L. (2015). Pathogenesis of hepatic encephalopathy: Role of ammonia and systemic inflammation. J. Clin. Exp. Hepatol..

[11] Sepehrinezhad A., Moghaddam N.G., Shayan N., Sahab Negah S. (2024). Correlation of ammonia and blood laboratory parameters with hepatic encephalopathy: A systematic review and meta-analysis. PLoS ONE.

[12] Zimmermann M., Reichert A.S. (2021). Rapid metabolic and bioenergetic adaptations of astrocytes under hyperammonemia—A novel perspective on hepatic encephalopathy. Biol. Chem..

[13] Jaffe A. (2020). Pathophysiology of Hepatic Encephalopathy. Clin. Liver Dis..

[14] Drews L., Zimmermann M., Westhoff P., Brilhaus D., Poss R.E., Bergmann L., Wiek C., Brenneisen P., Piekorz R.P., Mettler-Altmann T. (2020). Ammonia inhibits energy metabolism in astrocytes in a rapid and glutamate dehydrogenase 2-dependent manner. Dis. Model. Mech..

[15] Sepehrinezhad A., Zarifkar A., Namvar G., Shahbazi A., Williams R. (2020). Astrocyte swelling in hepatic encephalopathy: Molecular perspective of cytotoxic edema. Metab. Brain Dis..

[16] Llansola M., Montoliu C., Agusti A., Hernandez-Rabaza V., Cabrera-Pastor A., Gomez-Gimenez B., Malaguarnera M., Dadsetan S., Belghiti M., Garcia-Garcia R. (2015). Interplay between glutamatergic and GABAergic neurotransmission alterations in cognitive and motor impairment in minimal hepatic encephalopathy. Neurochem. Int..

[17] Elkrief L., Houssel-Debry P., Ackermann O., Franchi-Abella S., Branchereau S., Valla D., Hillaire S., Dutheil D., Plessier A., Hernandez-Gea V. (2020). Portal cavernoma or chronic non cirrhotic extrahepatic portal vein obstruction. Clin. Res. Hepatol. Gastroenterol..

[18] Sharma P., Sharma B.C., Puri V., Sarin S.K. (2008). Minimal hepatic encephalopathy in patients with extrahepatic portal vein obstruction. Am. J. Gastroenterol..

[19] de Ville d.G.J., Clapuyt P., Otte J.B. (1992). Extrahilar mesenterico-left portal shunt to relieve extrahepatic portal hypertension after partial liver transplant. Transplantation.

[20] Sharif K., McKiernan P., de Ville d.G.J. (2010). Mesoportal bypass for extrahepatic portal vein obstruction in children: Close to a cure for most!. J. Pediatr. Surg..

[21] Castro Rodríguez J., Rodríguez Perálvarez M.L., Montero-Álvarez J.L. (2024). Diagnosis and management of Abernethy syndrome. Rev. Esp. Enferm. Dig..

[22] Franchi-Abella S., Gonzales E., Ackermann O., Branchereau S., Pariente D., Guérin F., International Registry of Congenital Portosystemic Shunt Members (2018). Congenital portosystemic shunts: Diagnosis and treatment. Abdom. Radiol..

[23] Gioia S., Nardelli S., Riggio O., Faccioli J., Ridola L. (2021). Cognitive Impairement in Non-Cirrhotic Portal Hypertension: Highlights on Physiopathology, Diagnosis and Management. J. Clin. Med..

[24] Meena B.L., Narayan SJ A., Sarin S.K. (2025). Hepatic encephalopathy in non-cirrhotic portal hypertension. Metab. Brain Dis..

[25] Nardone R., Taylor A.C., Höller Y., Brigo F., Lochner P., Trinka E. (2016). Minimal hepatic encephalopathy: A review. Neurosci. Res..

[26] D’Antiga L., Dacchille P., Boniver C., Poledri S., Schiff S., Zancan L., Amodio P. (2014). Clues for minimal hepatic encephalopathy in children with noncirrhotic portal hypertension. J. Pediatr. Gastroenterol. Nutr..

[27] Srivastava A., Yadav S.K., Lal R., Yachha S.K., Thomas M.A., Saraswat V.A., Gupta R.K. (2010). Effect of surgical portosystemic shunt on prevalence of minimal hepatic encephalopathy in children with extrahepatic portal venous obstruction: Assessment by magnetic resonance imaging and psychometry. J. Pediatr. Gastroenterol. Nutr..

[28] Pujol A., Graus F., Peri J., Mercader J.M., Rimola A. (1991). Hyperintensity in the globus pallidus on T1-weighted and inversion-recovery MRI: A possible marker of advanced liver disease. Neurology.

[29] Montoliu C., Urios A., Forn C., Garcia-Panach J., Avila C., Gimenez-Garzo C., Wassel A., Serra M.A., Giner-Duran R., Gonzalez O. (2014). Reduced white matter microstructural integrity correlates with cognitive deficits in minimal hepatic encephalopathy. Gut.

[30] Yadav S.K., Srivastava A., Srivastava A., Thomas M.A., Agarwal J., Pandey C.M., Lal R., Yachha S.K., Saraswat V.A., Gupta R.K. (2010). Encephalopathy assessment in children with extra-hepatic portal vein obstruction with MR, psychometry and critical flicker frequency. J. Hepatol..

[31] Wang Y., Yang L., Shang Y., Huang Y., Ju C., Zheng H., Zhao W., Liu J. (2025). Identifying Minimal Hepatic Encephalopathy: A New Perspective from Magnetic Resonance Imaging. J. Magn. Reson. Imaging.

[32] Yadav S.K., Goel A., Saraswat V.A., Thomas M.A., Wang E., Marincola F.M., Haris M., Gupta R.K. (2016). Evaluation of cognitivity, proinflammatory cytokines, and brain magnetic resonance imaging in minimal hepatic encephalopathy induced by cirrhosis and extrahepatic portal vein obstruction. J. Gastroenterol. Hepatol..

[33] Rudler M., Weiss N., Perlbarg V., Mallet M., Tripon S., Valabregue R., Marjańska M., Cluzel P., Galanaud D., Thabut D. (2018). Combined diffusion tensor imaging and magnetic resonance spectroscopy to predict neurological outcome before transjugular intrahepatic portosystemic shunt. Aliment Pharmacol. Ther..

[34] Qi R., Zhang L.J., Luo S., Ke J., Kong X., Xu Q., Liu C., Lu H., Lu G.M. (2014). Default mode network functional connectivity: A promising biomarker for diagnosing minimal hepatic encephalopathy: CONSORT-compliant article. Medicine.

[35] Patel A., Kern M., Babaei A., Samuel E.A., Siwiec R.M., Chen G., Saeian K., Li S.J., Shaker R. (2013). Objective diagnosis of minimal hepatic encephalopathy (MHE) by analysis of brain resting state functional connectivity. Gastroenterology.

[36] Elam J.S., Glasser M.F., Harms M.P., Sotiropoulos S.N., Andersson J.L., Burgess G.C., Curtiss S.W., Oostenveld R., Larson-Prior L.J., Schoffelen J.M. (2021). The Human Connectome Project: A retrospective. Neuroimage.

[37] Chiu B., Superina R.A. (2006). Encephalopathy caused by a splenorenal shunt can be reversed by performing a mesenteric-to-left portal vein bypass. J. Pediatr. Surg..

[38] Mack C.L., Zelko F.A., Lokar J., Superina R., Alonso E.M., Blei A.T., Whitington P.F. (2006). Surgically restoring portal blood flow to the liver in children with primary extrahepatic portal vein thrombosis improves fluid neurocognitive ability. Pediatrics.

[39] Nieto-Castanon A., Whitfield-Gabrieli S. (2022). CONN Functional Connectivity Toolbox: RRID SCR_009550, Release 22.

[40] Behzadi Y., Restom K., Liau J., Liu T.T. (2007). A component based noise correction method (CompCor) for BOLD and perfusion based fMRI. Neuroimage.

[41] Sparacia G., Parla G., Mamone G., Caruso M., Torregrossa F., Grasso G. (2021). Resting-State Functional Magnetic Resonance Imaging for Surgical Neuro-Oncology Planning: Towards a Standardization in Clinical Settings. Brain Sci..

[42] Nichols T.E., Holmes A.P. (2002). Nonparametric permutation tests for functional neuroimaging: A primer with examples. Hum. Brain Mapp..

[43] Smith S.M., Nichols T.E. (2009). Threshold-free cluster enhancement: Addressing problems of smoothing, threshold dependence and localisation in cluster inference. Neuroimage.

[44] Salimi-Khorshidi G., Smith S.M., Nichols T.E. (2011). Adjusting the effect of non stationarity in cluster-based and TFCE inference. Neuroimage.

[45] Sparacino L., Faes L., Mijatović G., Parla G., Lo Re V., Miraglia R., de Ville de Goyet J., Sparacia G. (2023). Statistical Approaches to Identify Pairwise and High-Order Brain Functional Connectivity Signatures on a Single-Subject Basis. Life.

[46] Glickman M.E., Rao S.R., Schultz M.R. (2014). False discovery rate control is a recommended alternative to Bonferroni-type adjustments in health studies. J. Clin. Epidemiol..

[47] Llansola M., Izquierdo-Altarejos P., Montoliu C., Mincheva G., Palomares-Rodriguez A., Pedrosa M.A., Arenas Y.M., Felipo V. (2024). Role of peripheral inflammation in minimal hepatic encephalopathy. Metab. Brain Dis..

[48] Kibble H., Shawcross D.L. (2023). The microbiome in portal hypertension. Clin. Liver Dis..

[49] Lombardi M., Troisi J., Motta B.M., Torre P., Masarone M., Persico M. (2024). Gut-Liver Axis Dysregulation in Portal Hypertension: Emerging Frontiers. Nutrients.

[50] Zhang A., Wang J., Ji F., Qin B., Geng J., Kong G., Li Z. (2022). Improvement of gut microbiome and intestinal permeability following splenectomy plus pericardial devascularization in hepatitis B virus-related cirrhotic portal hypertension. Front. Immunol..

[51] Gitto S., Vizzutti F., Baldi S., Campani C., Navari N., Falcini M., Venturi G., Montanari S., Roccarina D., Arena U. (2023). Transjugular intrahepatic Porto-systemic shunt positively influences the composition and metabolic functions of the gut microbiota in cirrhotic patients. Dig. Liver Dis..

[52] Jiang W., Zhou Z., Li G., Yin W., Wu Z., Wang L., Ghanbari M., Li G., Yap P.T., Howell B.R. (2023). Mapping the evolution of regional brain network efficiency and its association with cognitive abilities during the first twenty-eight months of life. Dev. Cogn. Neurosci..

[53] Gozdas E., Holland S.K., Altaye M., CMIND Authorship Consortium (2019). Developmental changes in functional brain networks from birth through adolescence. Hum. Brain Mapp..

[54] Lillegard J.B., Hanna A.M., McKenzie T.J., Moir C.R., Ishitani M.B., Nagorney D.M. (2010). A single-institution review of portosystemic shunts in children: An ongoing discussion. HPB Surg..

